# Evolution of primary care databases in UK: a scientometric analysis of research output

**DOI:** 10.1136/bmjopen-2016-012785

**Published:** 2016-10-11

**Authors:** Paraskevas Vezyridis, Stephen Timmons

**Affiliations:** Centre for Health Innovation, Leadership and Learning (CHILL), Nottingham University Business School, Nottingham, UK

**Keywords:** scientometrics, PRIMARY CARE, electronic patient records, STATISTICS & RESEARCH METHODS

## Abstract

**Objective:**

To identify publication and citation trends, most productive institutions and countries, top journals, most cited articles and authorship networks from articles that used and analysed data from primary care databases (CPRD, THIN, QResearch) of pseudonymised electronic health records (EHRs) in UK.

**Methods:**

Descriptive statistics and scientometric tools were used to analyse a SCOPUS data set of 1891 articles. Open access software was used to extract networks from the data set (Table2Net), visualise and analyse coauthorship networks of scholars and countries (Gephi) and density maps (VOSviewer) of research topics co-occurrence and journal cocitation.

**Results:**

Research output increased overall at a yearly rate of 18.65%. While medicine is the main field of research, studies in more specialised areas include biochemistry and pharmacology. Researchers from UK, USA and Spanish institutions have published the most papers. Most of the journals that publish this type of research and most cited papers come from UK and USA. Authorship varied between 3 and 6 authors. Keyword analyses show that smoking, diabetes, cardiovascular diseases and mental illnesses, as well as medication that can treat such medical conditions, such as non-steroid anti-inflammatory agents, insulin and antidepressants constitute the main topics of research. Coauthorship network analyses show that lead scientists, directors or founders of these databases are, to various degrees, at the centre of clusters in this scientific community.

**Conclusions:**

There is a considerable increase of publications in primary care research from EHRs. The UK has been well placed at the centre of an expanding global scientific community, facilitating international collaborations and bringing together international expertise in medicine, biochemical and pharmaceutical research.

**Strengths and limitations of this study**

- First study to perform a scientometric analysis of research output from primary care databases of electronic patient records.
- We analysed articles published from 1995 to 2015 in order to explore the historical breadth and growth of this type of research.
- The analysis is limited to articles and structured data retrieved from the Scopus database.
- Some latest articles and related citations might not have appeared in Scopus when the data set was extracted.

## Introduction

Big data (analytics) refer to the aggregation and interrogation of—high volume, high velocity, high variety—data sets so as to reveal new, non-obvious, information and patterns.^1^ This field is advancing because of technological and scientific developments in information infrastructure and digitisation.^2^ For governments, opening up the data sets states hold about their citizens is believed to have, through computational and algorithmic analyses, a disruptive and transformative effect on knowledge.^3^ In the UK, big (open) data have been at the forefront of research activity and policymaking. Termed as one of the eight great technologies,^4^ UK has embraced the big (open) data movement more than many other developed countries (eg, USA, Australia, France).^3^ One area of particular relevance to big data analytics is healthcare.

In UK, the National Health Service (NHS) is organised around primary care and, unless there is an accident or emergency, whenever citizens would like to use the NHS they have to go through their primary care physician, known in the UK as a general practitioner (GP). From there, they can be referred to a specialist at a hospital if necessary. Secondary care clinicians can then feedback information to GPs. Since the vast majority of the population (98%) is registered with a general practice, GPs act not only as the main gatekeepers for the NHS but also as important custodians of a longitudinal electronic health record (EHR).^5^ There are now many ongoing primary care databases of anonymised patient records in UK that can be used for healthcare research. These population-based databases contain data originating from routine general practice. Some newly established databases and research platforms of linked EHRs include ResearchOne^6^ and CALIBER.^7^ While there are more than 9600 general practices in UK that could potentially contribute data to these databases,^8^ it is usually 6–10% of these practices that do so.

Such databases are usually used for cross-sectional surveys, case–control or cohort studies and for epidemiological, drug safety, clinical and healthcare usage research purposes. They rely heavily on individual general practices voluntarily contributing data via the propriety clinical systems they use to maintain these patient records. The records are usually anonymised or pseudoanonymised at source by allocating a unique number to each patient to allow for the updates of the records as well as for their linkage to other data sets, such as national mortality, national cancer registration and hospital records as well as with socioeconomic, ethnicity and environmental data sets. Access to these data sets is usually granted after scientific and ethics review and can be tailored to customer requirements. In this study, we examined the research output of three such databases that are well established in the research community and have contributed to a substantial number of scientific studies and publications. These are the Clinical Practice Research Datalink (CPRD),^9^ The Health Improvement Network (THIN)^10^ and QResearch.^11^

The CPRD (formerly known as the General Practice Research Database) is a not-for-profit research service funded by the NHS National Institute for Health Research (NIHR) and the Medicines and Healthcare products Regulatory Agency (MHRA). It is owned by the UK Department of Health and contains the records of 11 million patients (4.4 million active) from 674 general practices.^5^ There is a service cost associated with the preparation of the requested data. Unlike the other services described below, CPRD does not extract data from a particular propriety clinical system. Any general practice can contribute data after a data sharing agreement with the software supplier. Importantly, it is the only database accessible online.^12^ The THIN database contains the health records of 12 million patients from around 600 general practices that use the Vision clinical system by In Practice Systems (INPS). IMS Health can provide access to data, for example, via a yearly sublicense to an academic institution. THIN is the only database that can provide access to data for for-profit companies. QResearch is a research service located at the University of Nottingham. Its database contains the health records of 18 million patients from 1000 general practices that use the EMIS clinical system. Only academics employed by a UK university can have (in site) access only to a sample of the data set (maximum 100 000 patients) that is sufficient to answer a specific research question or hypothesis. As this research service is not-for-profit and entirely self-funded, there is usually a fee to be paid to cover the cost of the data extraction.

The strengths of these databases lie in their size, breadth, representativeness of the UK population, long-term follow-up and data quality.^5^ They include good information on morbidity and lifestyle, prescribing, preventive care, current standards of care and interpractice variation.^13^ Since they are continually (and automatically) updated, they are ideal for researchers to discover and monitor healthcare trends as well as the effectiveness of new interventions and treatments, with minimum cost. They are increasingly linked to secondary care and mortality data sets. In contrast, their weaknesses include the fact that data are extracted from propriety clinical systems developed for patient management and not for healthcare research. There are issues of missing data (eg, from healthy patients), variable definitions for diagnoses and incomplete secondary care data (eg, hospital admissions). Wider health data (eg, treatment adherence, over the counter medication) and data about subpopulations (eg, prisoners, homeless people, refugees, travellers) are not captured adequately.^5^ Information governance and informed consent procedures around the data sharing of EHRs for research are still considered complex.^14^ These databases also require considerable clinical and scientific expertise, as well as technical capacity in data management to support research. When selecting a particular data resource for an observational study, researchers have to consider several other factors, such as the population covered and its geographical distribution, data capture and latency, linkage with other resources, privacy and security, quality and validation.^15^

Nonetheless, these databases are highly regarded within the research community since they have proved their value in helping researchers reach definitive answers in various healthcare debates of considerable public interest, particularly where other types of research have produced contradictory evidence. For example, in 2004 researchers from UK and Canada proved beyond doubt that measles–mumps–rubella (MMR) vaccination is not associated with autism in children.^16^ In contrast to expensive, time-consuming, and unrepresentative (of the population) traditional randomised trials,^17^ large-scale and randomised observational (comparative) evaluations of treatments and medications are minimally obtrusive for clinicians and patients and can support faster turnaround times for pragmatic evidence useful in clinical practice.^18^

The aim of this study was to perform a scientometric analysis of articles, published from 1995 to 2015, which have used data from at least one of these primary care databases. This empirical, semiautomated, method of quantitatively analysing a large number of publications provides a reliable and objective examination of the current status and trends as well as the structure and dynamics of this scientific field.^19^ ^20^ In this way, policymakers, research funding bodies but most importantly new researchers entering this field can have a general overview of its knowledge base and an indication of what kind of network features, research activities and topics of interest are driving it.^20^ ^21^ To the best of our knowledge, this is the first study of a systematic mapping of primary care databases research output.

## Methods

In this study, Elsevier's Scopus database (http://www.scopus.com) was selected as the source of structured data on articles. This database covers more scientific articles than other databases (eg, Thomson Reuters Web of Science) and has the advantage of providing advance export functionality of structured data, including full citation information, abstracts, keywords and references. On 30 October 2015, we searched, using the document search functionality, for all articles containing the terms ‘General Practice Research Database (GPRD)’ OR ‘Clinical Practice Research Datalink (CPRD)’ OR ‘The Health Improvement Network (THIN)’ OR ‘QResearch’ in article title, abstract and keywords.

The results were then limited to *articles*, *articles in press*, *conference papers*, *reviews*, *book chapters* and *short surveys. Notes*, *letters*, *editorials* and *errata* were excluded from the analysis. From there, we compared the resulted records with the bibliographic lists maintained by these databases^22–24^ so as to include articles that could not be retrieved using the above search queries. Data cleansing included the removal of duplicate records and records that were missing essential information for the analysis (eg, article title, journal). The fields of *authors*, *year*, *source title*, *affiliations*, *author keywords* and *document type* were used for the analysis.

The final bibliography retrieved from Scopus was imported to Table 2 Net^25^ to extract networks of authors and contributing countries. It was then imported to Gephi^26^ where the ForceAtlas 2 algorithm^27^ was used to visualise the structural proximities for the communities of authors and contributing countries. The VOSviewer (V.1.6.3)^28^ software was used to visualise bibliometric networks and densities^29^ of frequent terms and journals. All other statistical analyses were performed using Microsoft Excel. We used the Journal Citation Reports (JCR) Science Edition 2014 to extract impact factor values for the identified journal titles.

## Results

A total of 1891 papers from 1995 to 2015 were included in this bibliometric and scientometric analysis. The results are presented below.

### Publication and citation trends

The literature related to the 3 primary care databases in England increased gradually from 7 papers in 1995 to 171 in 2015 (table 1). We estimated their compound annual growth rate (CAGR), for the years 1995–2014, to be 18.65%. The vast majority of papers were published in English across 425 different sources (16.76% CAGR for 1995–2014). In total, these papers have already been cited 73 929 times. There is, however, a small percentage of 1.16% (n=163) papers that have not yet been cited yet. The average citation per year is ∼3.52.

**Table 1:** Distribution of scientific literature by year

Year	No. of papers	Per cent	No. of citations	No. of different sources
2015	171	9.04	255	107
2014	214	11.31	1188	114
2013	203	10.73	1934	123
2012	175	9.25	3050	99
2011	148	7.82	3900	100
2010	129	6.82	5232	81
2009	126	6.66	5341	79
2008	115	6.08	4757	73
2007	96	5.07	5232	65
2006	74	3.91	5943	56
2005	81	4.28	5839	56
2004	73	3.86	6411	49
2003	46	2.43	2998	37
2002	52	2.74	3832	37
2001	51	2.69	3770	38
2000	51	2.69	6334	35
1999	26	1.37	1594	21
1998	29	1.53	2582	20
1997	17	0.89	1560	13
1996	7	0.37	1137	7
1995	7	0.37	1040	6
Total	1891	100	73 929	425

We explored the distribution of publications by document type. This is presented in table 2 to identify the preferences of scholars using these databases in their research to share knowledge. The vast majority of scholars prefer to publish the findings of their research through journals, particularly as original articles (96.5%).

**Table 2:** Distribution of scientific literature by document type

Type	No. of papers	Per cent
Article	1825	96.5
Conference paper	18	0.95
Book chapter	4	0.21
Review	41	2.16
Short survey	3	0.15

Next, we analysed the distribution of papers based on the academic discipline in which they have been categorised by Scopus (table 3) and by which each paper may be attributed to more than one subject area.^30^ Since we analysed bibliographic data based on published research using primary care databases, it comes as no surprise that the vast majority of papers are under the *medicine* category. There is, however, a considerable number of papers (∼25%) under the categories *biochemistry, genetics and molecular biology* and *pharmacology, toxicology and pharmaceutics*, which indicates an emphasis on the use of these databases for the study of medications. It also indicates the potential interest in these databases from the pharmaceutical sector.

**Table 3:** Distribution of scientific literature by discipline

Subject	No. of papers	Per cent
Medicine	1838	97.2
Biochemistry, genetics and molecular biology	266	14.1
Pharmacology, toxicology and pharmaceutics	197	10.4
Neuroscience	78	4.1
Immunology and microbiology	70	3.7
Agricultural and biological sciences	53	2.8
Nursing	44	2.3
Psychology	21	1.1
Arts and humanities	13	0.7
Environmental science	10	0.5

### Most productive institutions and countries

**Figure 1:**
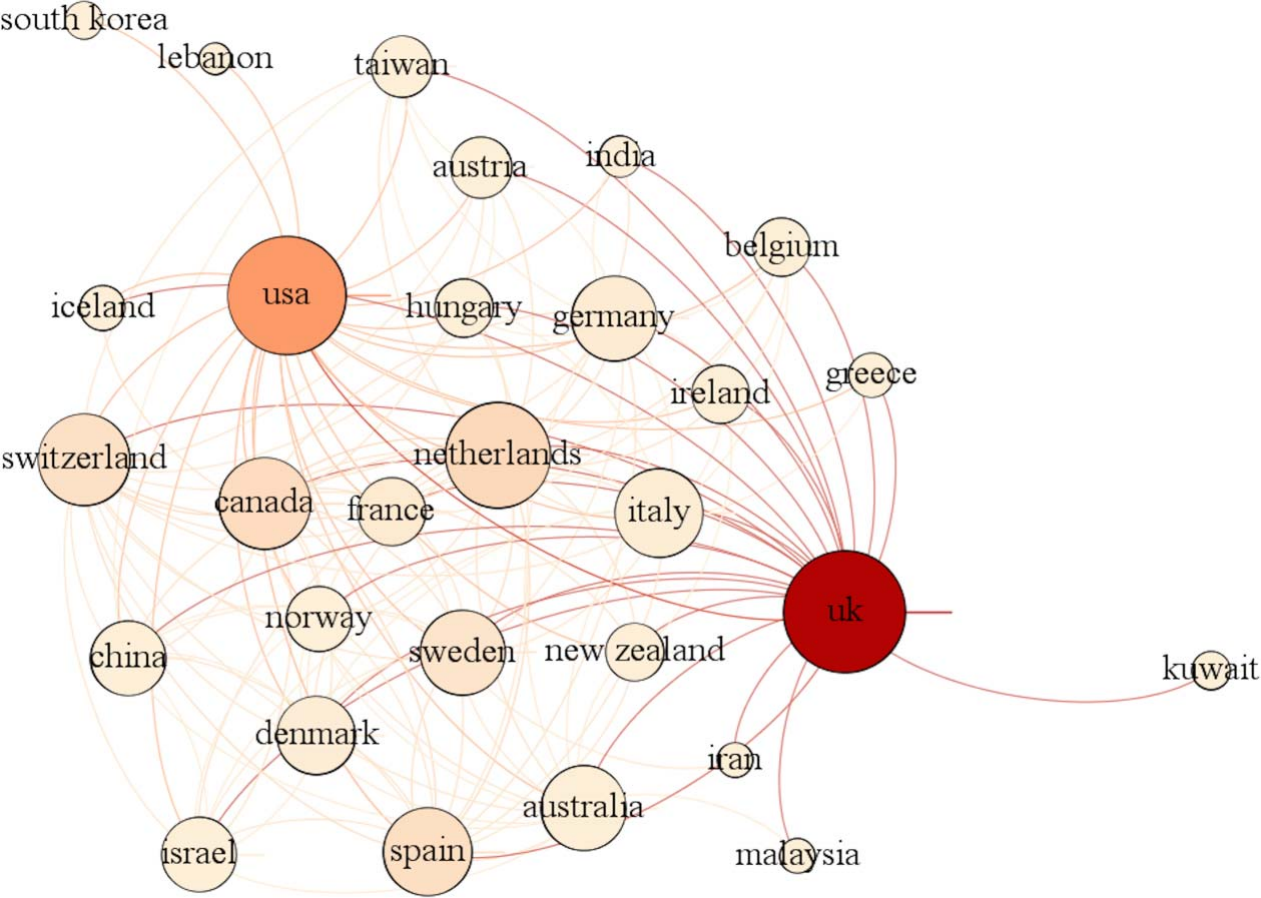
Network of contributing countries.

For a deeper insight into contribution patterns and scientific impact, we first identified the top 10 institutions (by number of papers) authors have used as affiliation and then we analysed citation patterns (table 4). We also analysed authors' affiliations based on the country of their institution. For this, each publication was assigned to its authors' respective affiliated countries so as to identify the network of multinational collaborations. The distribution of the top 10 contributing countries is presented in table 5. Finally, we visualised the network of contributing countries using Gephi. We ended up with a network of 29 nodes and 175 edges (figure 1). Each node represents a country, while its size denotes the country's degree and the colour the number of papers. The thickness of interconnected lines (edges) denotes the number of coauthored papers between the countries.

**Table 4:** Most productive institutions

Rank	Institution	No. of papers	Per cent	Total citations	Median (IQR)	Country
1	University of Nottingham	266	14.06	11 540	18 (6–44.75)	UK
2	Boston University	228	12.05	12 328	21.5 (6–57)	USA
3	Centro Espanol de Investigacion Farmacoepidemiologica (CEIFE)	163	8.62	8493	26 (7–59.5)	Spain
4	University College London	156	8.25	5226	14 (4.75–37)	UK
5	London School of Hygiene & Tropical Medicine	124	6.55	3696	14 (4–40.5)	UK
6	University of Utrecht	118	6.24	5067	17.5 (4–48.75)	The Netherlands
7	University of Pennsylvania	110	5.81	7508	24.5 (10–64.25)	USA
8	Medicines and Healthcare Products Regulatory Agency	94	4.97	2623	14 (4–33.25)	UK
9	King's College London	92	4.86	2221	13 (4–32.75)	UK
10	University of Oxford	86	4.54	1806	9.5 (3–23.25)	UK

**Table 5:** Top contributing countries

Rank	Country	No. of papers	Per cent
1	UK	1202	63.56
2	USA	563	29.77
3	Spain	192	10.15
4	Netherlands	164	8.67
5	Switzerland	115	6.08
6	Canada	112	5.92
7	Sweden	106	5.60
8	Germany	64	3.38
9	France	51	2.69
10	Italy	36	1.90

The majority of the most productive institutions are universities. Top universities include the University of Nottingham, Boston University, University College London (UCL), the London School of Hygiene & Tropical Medicine and the University of Utrecht. Apart from these academic institutions, a research unit in Spain (CEIFE) and the MHRA in the UK are involved in primary care databases-based research. In this table, we also report the medians along with the IQRs. From this, it seems that scholars from CEIFE, University of Pennsylvania and Boston University are coauthors in publications that are highly cited compared to the other institutions in this list. Switzerland, Canada, Sweden, Germany, France and Italy had no institution among the top 10 list, although they were ranked among the top 10 productive countries. Most papers are published by scholars from UK (63.56%), followed by the USA and Spain. With the exception of USA and Canada, most of the productive countries are in Europe. What is particularly interesting in these two tables is that scholars in institutions from the USA and Spain produce not only a great number of publications but also publications that are widely recognised by this scientific community in terms of citations.

Taking into account the measurements of *weighted degree*, *clustering*, *eigenvector centrality* and *betweenness centrality* (table 6), we observe that once again the UK, followed by USA, is placed at the centre of this scientific community. With the highest degrees of all measurements, institutions from this country are the most well-connected and authoritative ones, facilitating the linking between institutions in other countries.

**Table 6:** Top countries by centrality

Rank	Country	Occurrences	Weighted degree	Page rank	Eigen centrality	Closeness centrality	Betweenness centrality
1	UK	5770	27.0	0.062	1.0	0.933	0.250
2	USA	2506	26.0	0.060	0.98	0.903	0.229
3	Netherlands	666	22.0	0.047	0.95	0.8	0.049
4	Canada	567	18.0	0.039	0.83	0.717	0.024
5	Switzerland	409	18.0	0.039	0.85	0.717	0.015
6	Italy	85	17.0	0.037	0.78	0.7	0.024
7	Spain	481	17.0	0.037	0.82	0.7	0.011
8	Australia	37	16.0	0.036	0.76	0.682	0.024
9	Sweden	315	16.0	0.036	0.77	0.682	0.023
10	Germany	121	16.0	0.036	0.71	0.682	0.020

### Top journals

In table 7, we identify the top 10 journals where most research is published. Six of these journals are published by a UK publisher, and the rest are published in the USA. The journal *Pharmacoepidemiology and Drug Safety* features at the top of list, followed by the *British Journal of Clinical Pharmacology* and *Pharmacotherapy*, which signifies the focus of research, produced from these primary care databases, on the safe use of medication. This focus can also be seen in table 3, where (apart from medicine) most papers are published in the fields of *biochemistry, genetics and molecular biology* and *pharmacology, toxicology and pharmaceutics* (Scopus classification), but also in table 5, where most of the top-cited papers refer to potential risk for particular complications/conditions from the use of specific medication. More specialised journals, such as *Diabetes Care* and *Annals of the Rheumatic Diseases*, are also featured in this list, indicating a particular focus of research activity in specific spectrums of diseases.

**Table 7:** Top journals of published research

Rank	Journal name	No. of papers	Per cent	Publisher	Impact factor	Open access	Country
1	*Pharmacoepidemiology and Drug Safety*	115	6.08	Wiley	2.939	Hybrid	UK
2	*British Medical Journal*	100	5.28	BMJ Publishing Group	17.445	Full	UK
3	*British Journal of General Practice*	67	3.54	Royal College of General Practitioners	2.294	Full	UK
4	*British Journal of Clinical Pharmacology*	57	3.01	Wiley	3.878	Hybrid	UK
5	*PLoS One*	51	2.69	Public Library of Science	3.234	Full	USA
6	*BMJ Open*	41	2.16	BMJ Publishing Group	2.271	Full	UK
7	*Pharmacotherapy*	34	1.79	Wiley	2.662	Hybrid	USA
8	*Diabetes Care*	30	1.58	American Diabetes Association	8.420	Hybrid	USA
9	*Epidemiology*	24	1.26	Wolters Kluwer	6.196	Hybrid	USA
10	*Annals of the Rheumatic Diseases*	23	1.21	BMJ Publishing Group	10.377	Hybrid	UK

Four journals in this list are open access (*BMJ*, *British Journal of General Practice*, *PLoS One*, *BMJ Open*), which greatly facilitates the sharing of knowledge without limitations. The *BMJ* enjoys widespread recognition of the high quality of its published studies, as indicated by the high impact factor. The rest are behind a pay wall but offer authors an open access option to publish their research (hybrid access). An extra column with the Impact Factors of these top 10 journals from the 2014 JCR was also added in the table.

**Figure 2:**
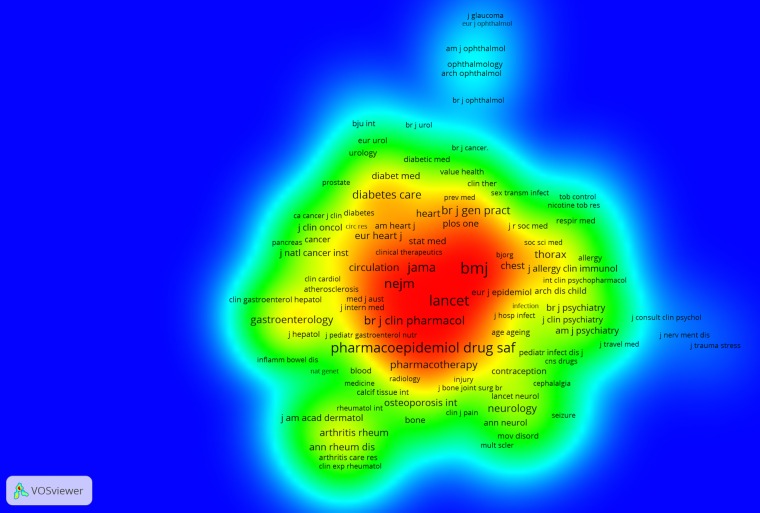
Journal cocitation analysis.

What is also of particular importance in terms of scientific impact is that the 10 most cited papers identified (see table 8) have not been published in journals in this list. However, by performing a (full counting) analysis of cocitation links in VOSviewer (figure 2) for journals cited in the Scopus data set (minimum number of citations=10) we see that most of this list is represented here (blue—lowest density, red—highest density).

**Table 8:** Most cited papers

Rank	Authors/title	Year	Country	Journal	Impact factor	Citations	Open access
1	Jick H, Zornberg GL, Jick SS, Seshadri S, Drachman DA	2000	USA	*Lancet*	45.217	1322	No
	Statins and the risk of dementia						
2	Gelfand JM, Neimann AL, Shin DB, Wang X, Margolis DJ, Troxel AB	2006	USA	*Journal of the American Medical Association*	35.289	854	Yes
	Risk of myocardial infarction in patients with psoriasis						
3	Van Staa TP, Leufkens HGM, Abenhaim L, Zhang B, Cooper C	2000	UK	*Journal of Bone and Mineral Research*	6.832	796	Yes
	Use of oral corticosteroids and risk of fractures						
4	Henry D, Lim LLY, Rodriguez LAG, Perez Gutthann S, Carson JL, Griffin M, Savage R Logan R, Moride Y, Hawkey C, Hill S, Fries JT	1996	Australia, Spain, USA, New Zealand, UK	*British Medical Journal*	17.445	688	Yes
	Variability in risk of gastrointestinal complications with individual non-steroidal anti-inflammatory drugs: Results of a collaborative meta-analysis						
5	Yang YX, Lewis JD, Epstein S, Metz DC	2006	USA	*Journal of the American Medical Association*	35.289	637	Yes
	Long-term proton pump inhibitor therapy and risk of hip fracture						
6	Currie CJ, Poole CD, Gale EAM	2009	UK	*Diabetologia*	6.671	629	Yes
	The influence of glucose-lowering therapies on cancer risk in type 2 diabetes						
7	Jick H, Jick SS, Gurewich V, Myers MW, Vasilakis C	1995	USA, UK	*Lancet*	45.217	612	No
	Risk of idiopathic cardiovascular death and nonfatal venous thromboembolism in women using oral contraceptives with differing progestagen components						
8	Dial S, Delaney JAC, Barkun AN, Suissa S	2005	Canada	*Journal of the American Medical Association*	35.289	582	Yes
	Use of gastric acid-suppressive agents and the risk of community-acquired *Clostridium difficile*-associated disease						
9	Smeeth L, Thomas SL, Hall AJ, Hubbard R, Farrington P, Vallance P	2004	UK	*New England Journal of Medicine*	55.873	546	Yes
	Risk of myocardial infarction and stroke after acute infection or vaccination						
10	Neimann AL, Shin DB, Wang X, Margolis DJ, Troxel AB, Gelfand JM	2006	USA	*Journal of the American Academy of Dermatology*	4.449	528	No
	Prevalence of cardiovascular risk factors in patients with psoriasis						

### Most cited papers

Next, we focused on the top 10 papers^31–40^ and calculated the total count of citations for each paper (table 8) for the period 1995–2015. Citations totalled 7194 (1.02%) of all citations in this data set. It seems that these studies in dementia, psoriasis, fractures, cardiovascular diseases and gastrointestinal complications in relation to certain medications have been of great interest in this scientific community. The majority of the top 10 most cited papers (60%) are open access at the publisher's website and can be freely read by anyone.

Of the 10 papers, 8 are single country papers, while none were singled authored. Again, the USA has a considerable presence in this list, producing papers that are highly cited. In addition, many of the highly productive authors identified (table 10) were also found in this list.

### Authorship patterns and networks

Authorship distributions varied from single to a maximum of 155 authors—for a study about the feasibility of international collaboration to evaluate, based on a common protocol, the risk of Guillain–Barré syndrome following pH1N1 vaccination.^41^ In total, there were 9385 authors involved in the 1981 papers during 1995–2015. In table 9, we can see that more than three-quarters of all papers were published by three or more authors. Only 1% of papers were written by a single author, while five papers did not have any authorship details. Almost a quarter of all papers was published by four authors, which have been widely cited across this scientific community. This indicates the high degree of expert collaboration in this field, that is necessary in analysing millions of primary care records.

**Table 9:** Coauthorship distribution

Rank	No. of authors	No. of papers	Per cent	Citations	Per cent
1	4	460	24.33	16 437	22.23
2	5	383	20.25	16 071	21.74
3	3	310	16.39	12 209	16.51
4	6	252	13.33	12 084	16.35
5	2	145	7.67	7159	9.68
6	7	123	6.50	3798	5.14
7	8	88	4.65	2914	3.94
8	>11	48	2.54	1792	2.42
9	9	28	1.48	617	0.83
10	10	25	1.32	412	0.56
11	1	19	1.00	436	0.59

Table 10 provides the ranking of the top 10 scholars, first, in terms of research productivity based on the overall number of coauthored papers. While, generally, most scholars are from the UK, the Director of the Spanish Centre for Pharmacoepidemiologic Research (CEIFE)^42^ is the scholar with the most published research from these primary care databases. Also, there are researchers in this field who do not necessarily come from the academic environment. The pharmaceutical sector is actively involved in knowledge production from electronic primary care records.

**Table 10:** Most productive authors

Rank	Author	Affiliation	Country	No. of papers	Weighted degree	Clustering	Eigen centrality	Closeness centrality	Betweenness centrality
1	Rodriguez LAG	Spanish Centre for Pharmacoepidemiologic Research (CEIFE)	Spain	166	82.0	0.082	0.346	0.337	0.073
2	Jick SS	Boston University	USA	142	90.0	0.066	0.327	0.329	0.078
3	Van Staa TP	University of Manchester	UK	115	144.0	0.063	1.0	0.403	0.217
4	Jick H	Boston University	USA	98	55.0	0.098	0.217	0.322	0.032
5	Meier CR	University of Basel	Switzerland	94	54.0	0.116	0.232	0.294	0.014
6	Hubbard R	University of Nottingham	UK	86	82.0	0.092	0.441	0.359	0.067
7	Smeeth L	London School of Hygiene & Tropical Medicine	UK	79	89.0	0.101	0.578	0.366	0.067
8	Hippisley-Cox J	University of Nottingham	UK	72	57.0	0.144	0.244	0.325	0.065
9	Johansson S	AstraZeneca	Sweden	70	46.0	0.218	0.267	0.310	0.012
10	Cooper C	University of Southampton	UK	65	67.0	0.139	0.414	0.342	0.035
	West J	University of Nottingham	UK	65	42.0	0.213	0.230	0.310	0.012

**Figure 3:**
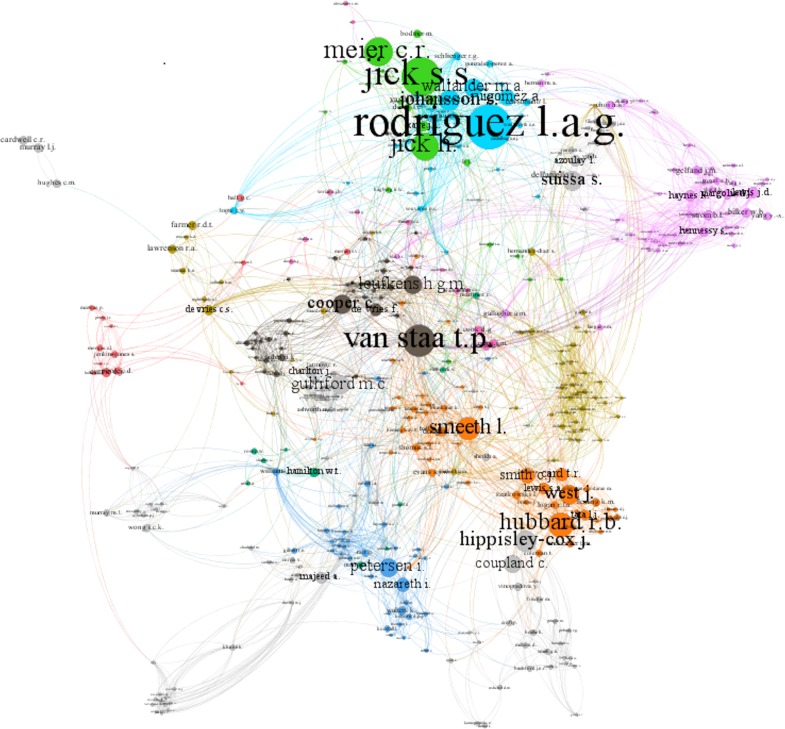
Clustered coauthor network.

Considering only those scholars who have coauthored at least two papers in this data set, the analysis suggested a network (figure 3) with 1261 nodes and 6186 edges. Here, each node represents an author, while its size denotes the number of author's papers. The interconnected lines (edges) denote the coauthored papers between those authors. For better visualisation, we limited the number of minimum degrees to 5 (maximum degrees=145). After a modularity measurement, to identify community structure,^43^ we observe some established collaborative teams (clusters with different colours) around specific and highly productive scholars in the analysis of data from primary care databases also found in table 10. We also observe a new (blue) cluster around the lead statistician for THIN^44^—one of the three primary care databases studied.

Taking into account the measurements of *weighted degree*, *clustering*, *eigenvector centrality* and *betweenness centrality* (table 10), results indicate a cluster placed at the centre of this scientific community. With the lowest degree of clustering and the highest degrees of all the other measurements, its prominent scholar is the most well-connected, facilitating, more than any other scholar, linking between other scientific clusters and scholars.

What is also particularly interesting is the fact that some of the scholars (and their institutions) in this list are affiliated, to a certain extent, to these databases, having served or currently acting as their founders, directors, lead scientists or members of their scientific committee.^45^ ^46^ For example, lead scientists from the Boston Collaborative Drug Surveillance Program in Boston University were among the first who developed the technical and scientific capacity of these databases in pharmacoepidemiological research.^47^ ^48^

### Research topics

**Figure 4:**
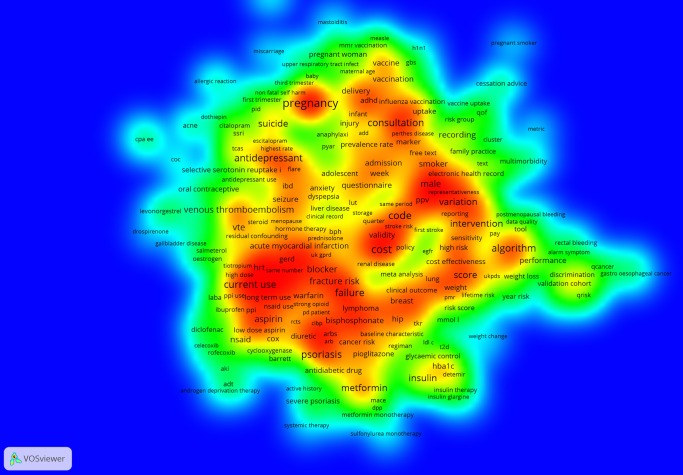
Term co-occurrence density map.

We conducted a keyword analysis to identify important topics of published research. For this, we first extracted from the bibliographic data set 5813 unique keywords as indexed by Scopus^30^ to base our analysis on more complete indexing information compared to authors' keywords. We retrieved the top 30 keywords for two specific categories: *medical conditions* and *medications/substances* (tables 11 and 12). Next, we created a (full counting of) term co-occurrence density map in VOSviewer (figure 4) by building a text corpus out of the title and abstract fields in the bibliographic data set (minimum number of a term occurrence=10). In this way, we were able to identify topics that not only appear more frequently in the literature, but that were also strongly related to each other, forming clusters of topics. Blue indicates a low density of terms and red indicates the highest density of terms. In many cases, the density map represents the frequency of indexed keywords in tables 11 and 12. Clearly, smoking, diabetes, cardiovascular diseases, mental illnesses, psoriasis, obesity, pregnancy and cancer as well as medication and substances that can treat these medical conditions, such as aspirin, insulin, antidepressants and non-steroid anti-inflammatory agents (NSAIDs), have been of great interest for scholars using EHRs in primary care.

**Table 11:** Top keywords: medical conditions

Rank	Keyword	Occurrences	Rank	Keyword	Occurrences
1	Smoking	328	16	Cardiovascular diseases	96
2	Diabetes mellitus	223	17	Myocardial infarction	94
3	Hypertension	223	18	Chronic obstructive lung disease	90
4	Non-insulin dependent diabetes mellitus	179	19	Heart failure	86
5	Depression	167	20	Cerebrovascular accident	85
6	Stroke	165	21	Rheumatoid arthritis	83
7	Asthma	158	22	Epilepsy	81
8	Diabetes mellitus, type 2	155	23	Breast cancer	78
9	Cancer risk	150	24	Fracture	75
10	Cardiovascular risk	147	25	Psoriasis	75
11	Cardiovascular disease	133	26	Gastrointestinal haemorrhage	69
12	Obesity	129	27	Hip fracture	68
13	Heart infarction	126	28	Osteoporosis	68
14	Pregnancy	125	29	Colorectal cancer	65
15	Ischaemic heart disease	104	30	Fractures, bone	65

**Table 12:** Top keywords: medications/substances

Rank	Keyword	Occurrences	Rank	Keyword	Occurrences
1	Non-steroid anti-inflammatory agent	182	16	Proton pump inhibitor	75
2	Acetylsalicylic acid	154	17	Warfarin	71
3	Metformin	150	18	Antidiabetic agent	69
4	Corticosteroid	143	19	Anticonvulsive agent	68
5	Hydroxymethylglutaryl coenzyme a reductase inhibitor	138	20	Serotonin uptake inhibitor	65
6	Insulin	133	21	Calcium channel blocking agent	64
7	Antidepressant agent	124	22	Antibacterial agents	62
8	β adrenergic receptor blocking agent	108	23	Hydroxymethylglutaryl-coA reductase inhibitors	62
9	Hypoglycemic agents	91	24	Oral antidiabetic agent	61
10	Anti-inflammatory agents, non-steroidal	90	25	Paracetamol	56
11	Dipeptidyl carboxypeptidase inhibitor	88	26	Diuretic agent	53
12	Antihypertensive agent	82	27	Ibuprofen	52
13	Neuroleptic agent	80	28	Simvastatin	52
14	Antibiotic agent	77	29	Tricyclic antidepressant agent	52
15	Hemoglobin A1c	75	30	Diclofenac	50

## Discussion

This study identified the leading institutions, countries, authors, journals and topics as well as their networks of published research that have used primary care databases in the UK to extract and analyse data from EHRs. There is a growing production of such papers which indicates the interest of a global and highly collaborative scientific community in this field and also the knowledge and insights that can be gained for healthcare improvement. Publication output increased from 7 papers in 1995 to 171 by October 2015 (18.65% CAGR for 1995–2014). It may be worth noting that by performing a similar, limited to the UK, search in Scopus for the same period and with the keyword ‘primary care’ we found a 10.83% CAGR, which shows the increase in research conducted from these databases outstrips the field more generally. The vast majority of publications (96.5%) were journal articles. While this research field can be located, generally, in medicine, biochemical and pharmaceutical developments seem to be equally important, aimed at addressing widespread medical conditions, such as diabetes, cardiovascular diseases, mental illnesses, smoking, obesity and cancer.

The UK has been well placed in this scientific field. This is partly due to the fact that there is now more than 30 years of data available in GP information systems.^49^ The investment in developing primary care databases from EHRs for research purposes has placed the country at the centre of a network of collaborations across the globe, bringing together international expertise for the analysis of ever-expanding and increasingly interlinked clinical data sets from primary, secondary and tertiary care. Access to these data sets has also allowed researchers and institutions from other countries to develop their own programmes of research to answer important clinical questions. Six of the most productive institutions are located in the UK, and 63.56% of publications were authored by scholars affiliated with this country, followed by the USA. Interestingly, the top institutions were not exclusively universities. Among them, we can find a research unit in Spain (CEIFE) and the executive agency in UK that funds and runs the CPRD database (MHRA), while one of the most productive scholars is affiliated with a pharmaceutical company. This signifies the great interest of various actors, from academic, governmental and private sectors, in research with primary care databases.

The geographical trend can also be observed from the location of journals with the most published papers. Six of the top 10 journals are published in the UK, followed by the USA. The journals with the most papers published included *Pharmacoepidemiology and Drug Safety*, *BMJ*, *British Journal of General Practice* and *British Journal of Clinical Pharmacology*, which signifies the great interest of scholars in using data from EHRs for pharmaceutical research. This is partially because one of the oldest sets of routine information collected by GP practices in the UK and made available by these databases is drug histories.^47^ Regarding restrictions on access to research outputs, only four journals in this list are fully open access. This may limit access to knowledge to researchers and members of the public that cannot afford subscription costs. Interestingly, it is the more established journals in medicine, such as *JAMA*, *Lancet* and *NEJM*, that have published some of the most cited papers in this bibliometric data set and enjoy a high level of cocitation activity.

Keyword analyses show that smoking, diabetes, cardiovascular diseases, mental illnesses, psoriasis, obesity, pregnancy and cancer constitute the main topics of research activity using EHRs in primary care. Often, this research concentrates on developing algorithms to identify risk of occurrence of a particular disease. Researchers are also interested in investigating medications that can treat these medical conditions, such as aspirin, other NSAIDs, insulin and antidepressants.

For the vast majority of publications, authorship varied between three and six authors, indicating widely collaborative, international, efforts to promote research in this field. Coauthorship network analyses showed that the lead scientists, directors and founders of these databases were found, to various degrees, at the centre of clusters in this scientific community, highlighting their invaluable contribution to knowledge production. As Azoulay *et al*^50^ have demonstrated in their study about eminent researchers and the vitality of a field, the development of coauthorship networks and clusters of collaborators in newly established scientific domains might be useful to boost research productivity. On the basis of each database's data access requirements, their established researchers appear to have a fundamental role in facilitating and promoting international collaborations for more researchers, institutions and countries. Importantly, they have a clear and in-depth understanding of the kind of research activities these databases can support in terms of data quality, structure and EHR coding practices. As these databases are expected to open up in the future to more stakeholders from various disciplines around health and as universities prepare to incorporate training in data science skills (eg, statistics, biomedical informatics, biology and medicine)^51^ into their clinical curricula, so as to nurture the next generation of clinical investigators,^52^ these established researchers could promote quality, reliable and ethically appropriate scientific research^53^ from complicated and highly contextual data sets.

Our study has the typical limitations of a scientometric study. We analysed articles published in a period of 20 years in order to explore the historical breadth and growth of research from electronic primary care records. However, this analysis is limited on structured data retrieved from one bibliometric database of peer-reviewed literature. Therefore, only articles published in journals in its index were analysed. Also, some of the latest articles and related citations might not have been retrieved at the time of the search, which might explain the decrease in the number of publications and citations particularly from 2010 onwards. It was beyond the scope of this quantitative study to assess the scientific quality and the socioeconomic impact of the large number of publications analysed here. These studies have deployed a range of study designs across many subfields of primary care research and with various research findings. Our main objective was restricted to assessing one aspect of academic impact and research quality, that is, patterns and trends in research outputs.^54^ Future research could focus on the wider academic and socioeconomic impact of these studies by examining the relationship between publications, citation patterns and collaborations with the development of new scientific methods in the field or of new medical products and healthcare services.

In conclusion, output of primary care research from EHRs has consistently increased since their development. The development of these databases in the UK has placed the country and affiliated academic institutions at the centre of an expanding global scientific community, facilitating international collaborations and bringing together international expertise in medicine, biochemical and pharmaceutical research.
